# Potassium fractions in black soil mediated by integrated nutrient management modules under maize-chickpea cropping sequence

**DOI:** 10.1371/journal.pone.0292221

**Published:** 2023-09-29

**Authors:** C. K. Dotaniya, B. L. Lakaria, Yogesh Sharma, B. P. Meena, R. H. Wanjari, A. O. Shirale, M. L. Dotaniya, Satish B. Aher, Priya Gurav, Pramod Jha, A. K. Biswas, S. R. Yadav, Kuldeep Kumar, R. K. Doutaniya, M. L. Reager, Manju Lata, R. C. Sanwal

**Affiliations:** 1 Department of Soil Science & Agricultural Chemistry, College of Agriculture, Bikaner, India; 2 ICAR- Indian Institute of Soil Science, Bhopal, India; 3 ICAR-Directorate of Rapeseed -Mustard Research, Bharatpur, India; 4 ICMR- National Institute for Research in Environmental Health, Bhopal, India; 5 ICAR-Indian Institute of Soil & Water Conservation, RC Kota, India; 6 Department of Agronomy, SKN College of Agriculture, Jobner, India; 7 University of Rajasthan, Jaipur, India; MVJ College of Engineering, INDIA

## Abstract

A field experiment was conducted at the Research Farm of the ICAR-Indian Institute of Soil Science, Bhopal (India) to study influence of different integrated nutrient management (INM) modules on soil potassium (K) fractions. The experiment comprised with twelve treatments laid out in randomized block design (RBD) with three replications under maize-chickpea cropping sequence. The treatments included general recommended dose (GRD), soil test crop response (STCR) dose; combinations of inorganic and organic inputs and only organic modules. The soil samples were collected at crop harvest and analyzed for various K fractions viz., water soluble-K, available-K, exchangeable-K, HNO_3_-K, lattice-K and total-K. The results indicated that potassium fractions were significantly (p = 0.05) affected by different treatments. Different INM modules significantly enhanced significantly K availability in soil. Among various INM modules studied, treatment 11 (application of 20 t ha^-1^ FYM in maize with 5 t ha^-1^ FYM every year in chickpea) proved most beneficial for improving the soil K fractions. Findings of this type are important for K fertilizer management during crop production in areas with low soil fertility.

## Introduction

The growing global population has compelled us to increase food production, despite our limited natural resources. In this context, developing countries like India, which is projected to have a population of 1.66 billion by 2050, will require approximately 333 million tonnes (mt) of food grain. Unfortunately, the quality and availability of these natural resources are on the declined [1]. Additionally, it has been observed that the increasing threat of insect pests, the incidence of diseases, poor nutrient levels, and limited availability of irrigation water are all exacerbated by the changing scenario of climate change, leading to a reduction in crop productivity. Soil health parameters are also diminishing, which has a significant impact on the potential yield of crops. Diminishing soil fertility stands out as a primary contributor directly impacting crop yields. The use of fertilizers plays a pivotal role in safeguarding soil fertility and bolstering productivity. In India’s arable regions, soil fertility often deteriorates due to the substantial nutrient requirements of crops and imprecise fertilizer application. Consequently, effective soil fertility management is imperative to guarantee productivity, nutritional security, and the long-term health and sustainability of the soil [2]. Specifically, inadequate utilization of potassium fertilizers emerges as a significant contributor to declining soil fertility and reduced crop productivity across various regions of India. Potassium (K) is a major constituent of the earth crust. On average, 2.6% of the Earth crust is made of K, making it the seventh most abundant element and fourth most abundant mineral nutrient in the lithosphere [3]. In soil, K exists in four different forms *viz*., soil solution-K, exchangeable-K, non-exchangeable-K and mineral-K. Soil K mainly exists as constituent of mineral structure and in fixed or non-exchangeable forms with minor portion as water soluble and exchangeable-K [1]. These forms of K are correlated with each other and a dynamic equilibrium exists among them [4]. Due to its activation of numerous enzymes, its role in maintaining water balance in plants is crucial for plant metabolism and development. It also contributes to protein synthesis, photosynthesis, fruit quality, disease and insect pest prevention, as well as cold and frost resilience. Depletion in the soil solution K is likely to shift the equilibrium in the direction to replenish it. The equilibrium reactions affect the level of soluble K at any time, and accordingly the amount of K available for plants [5].

The knowledge about different forms and availability of K is must while studying the response of crops to K because K supply to crop plants is a complex phenomenon involving relationships among various K fractions in soil. Plants utilize not only readily available K but also, at times, non-exchangeable and mineral potassium during crop growth. Potassium is released in soluble and exchangeable forms during the weathering of potassium-bearing minerals, and this release can occur at widely varying rates. Soil solution K is most mobile and prone to leaching in soils. Exchangeable and solution K are often considered as forms readily available to plant, while non-exchangeable and mineral K are slowly available forms [6]. Solution and exchangeable K are replenished by non-exchangeable K when the former are depleted by plant removal or leaching [7]. Potassium fertility status in Indian agricultural soils are categorized accordingly: 21%, 51%, and 28% as low, medium and high, respectively. About 72% of India’s agricultural area, representing 266 districts, needs immediate K fertilization [8].

Minimizing the need for K during crop production using organic inputs poses a significant challenge for farmers aiming to meet their organic sourcing requirements. The availability of farmyard manure (FYM) has been steadily declining over time. Another issue arises from organic sources containing lower quantities of essential plant nutrients, necessitating the application of larger volumes of organic materials to meet recommended fertilizer doses for crops. However, the addition of inorganic fertilizers over an extended period to fulfill the requirements of a specific nutrient also carries the risk of disrupting the balance of other plant nutrients in the soil. Furthermore, this practice can have adverse effects on the microbial population and disrupt the various cycles of plant nutrient availability in the soil. A promising solution involves the application of both organic and inorganic sources of potassium in maize and chickpea crop cultivation, which has been shown to improve the soil’s overall plant nutrient status. Integrated nutrient management (INM) improves soil fertility status of soil and enhance crop yield. INM improve the soil microbial population and diversity and mediate the plant nutrient dynamics in soil [9, 10]. Potassium in soil behaves in somewhat elusive manner in the sense that while the total K content in most soils is quite high, its availability is largely influenced by a number of soil and plant related factors.

In the above facts, a hypothesis was formulated to monitor the K different forms in INM under maize-chickpea sequence as dominated in this region. This study will be helped to apply the judicious application of organic and inorganic source of plant nutrients during the crop cultivation.

## Materials and methods

### Experiment site

A field experiment was conducted at the research farm of the ICAR-Indian Institute of Soil Science (ICAR-IISS), Bhopal, India. Geographically, the ICAR-IISS lies between 23°18’ N latitude and 77°24’ E longitudes. The elevation above mean sea level is 485 m. The climate of study site is sub-humid and in characterized by cool and dry winters, hot summer and humid monsoon with a mean annual rainfall of 1146 mm 75–80% of which is received during June to September. The mean potential evapo-transpiration is 1208 mm.

### Experiment details

The on-going research project on “Long-term evaluation of integrated plant nutrient supply modules for sustainable productivity in a Vertisol” was used for this investigation (Table 1). During initial 10 years soybean-wheat cropping system was used and there after it was replaced with maize–chickpea from 2012–13. Maize (Pro-Agro 4212)–Chickpea (JG-315), were grown with 20 and 70 kg ha^-1^ seed rate for maize–and chickpea with row to row and plant to plant spacing of 60 cm X 25 cm and 30 cm X 10 cm for maize and chickpea, respectively. Crops were grown as per standard cultural practices. The general recommended dose (GRD) of fertilizer application for these crops was 120-60-30 and 20-60-20 kg N, P_2_O_5_ and K_2_O, respectively for maize and chickpea. The plot size of the experiment was 20 X 5 sq meters.

**Table 1:** Treatment details in maize-chickpea cropping sequence in a Vertisol of central India.

Treatment	Maize	Chickpea
T_1_ (Control)	No Fertilizer/ Manure	No Fertilizer/ Manure
T_2_ (GRD)	120–60–30	20-60-20
T_3_ (STCR dose)	135-55-50 (5 t ha^-1^)	0-0-0 (1.5 t ha^-1^)
T_4_	75% NPK of T_3_	100% P only
T_5_	75% NPK of T_3_ + 5 t ha^-1^ FYM	100% P only
T_6_	75% NPK of T_3_ + 1 t ha^-1^ PM	100% P only
T_7_	75% NPK of T_3_ + 5 t ha^-1^ UC	100% P only
T_8_	75% NPK of T_3_ + MR mulch incorporated	100% P only + MR mulch
T_9_	1 t ha^-1^ PM + Gly 2 t ha^-1^ + MR mulch incorporated	100% P only + MR mulch
T_10_	5 t ha^-1^ FYM + Gly 2 t ha^-1^ + MR mulch incorporated	100% P only + MR mulch
T_11_	20 t ha^-1^ FYM (every season)	5 t ha^-1^ FYM (every season)
T_12_	75% NPK of T_3_ + 20 t ha^-1^ FYM (once in 4 years)	100% P only

GRD—General recommended dose (kg ha^-1)^, STCR—Soil test crop response dose, MR—Maize residues, FYM—Farm yard manure, PM—Poultry manure, UC—Urban compost, WR- Wheat Residue, Gly–Glyricidia

### Experimental soil

The physico-chemical properties experimental soil was analysed as per the method described in Singh *et al*. [11]. The soil of experimental site is classified as Vertisol (*Typic Haplusterts*) with smectite as the dominant clay mineral. Vertisols are churning heavy clay soils with a high proportion of swelling clays. These soils form deep wide cracks during summer season. The soil of the experimental site is clayey in texture with 25.2, 18 and 56.8 per cent of sand, silt and clay, respectively. The soil was medium in soil organic carbon (0.53%), low in available N (68.8 mg kg^-1^), medium in available P (12.8 mg kg^-1^) and high in available K (237 mg kg^-1^). The soil was normal in reaction (pH 7.76) and electrical conductivity (EC) was 0.48 dS m^-1^. DTPA extractable micronutrients Fe, Mn, Zn and Cu were measured 5.52, 9.44, 0.74 and 1.32 mg kg^-1^, respectively.

### Soil sampling, processing and determination of soil K fractions

Soil samples were collected from 0–15 cm depth after harvest of *winter* season crop from ten years old ongoing experiments; and were homogenized. Visible litter and roots were picked out from collected soil samples. The soil samples were air-dried at room temperature, ground and sieved through 2 mm sieve. The processed soil was stored in air tight plastic containers for further analysis of different K fractions. Different K fractions in soil sample viz., water soluble-K, available-K, exchangeable-K, HNO_3_-K, lattice-K and total-K were determined following standard methods. The water-soluble K was determined in 1:2.5 soil: water extract by shaking soil-water suspension for half an hour [12]. The soil available K was extracted from the soil using neutral normal ammonium acetate in 1:5 soil solution ratio and the K in extract was recorded using flame photometer [13]. The soil Exchangeable-K fraction was calculated as the difference between available K and water soluble K. The HNO_3_-K was determined by boiling nitric acid extraction method as outlined by Wood and Deturk [14]. The total- K content was determined by digesting the soil samples with hydrofluoric acid (HF) in a closed vessel [15]. The lattice-K was calculated by deducting HNO_3_-K from total K.

### Statistical analysis

The experiment comprised with twelve treatments laid out in a randomized block design (RBD) with three replications under maize-chickpea cropping sequence. All the measurements are the mean value of three separate replicates. Data was subjected to an analysis of variance. The mean values were grouped for comparisons and the least significant differences (LSD) among them were calculated at p < 0.05 confidence level using ANNOVA statistics as outlined by Gomez and Gomez [16].

## Results and discussion

### Soil K fraction under different INM modules

**Fig 1:**
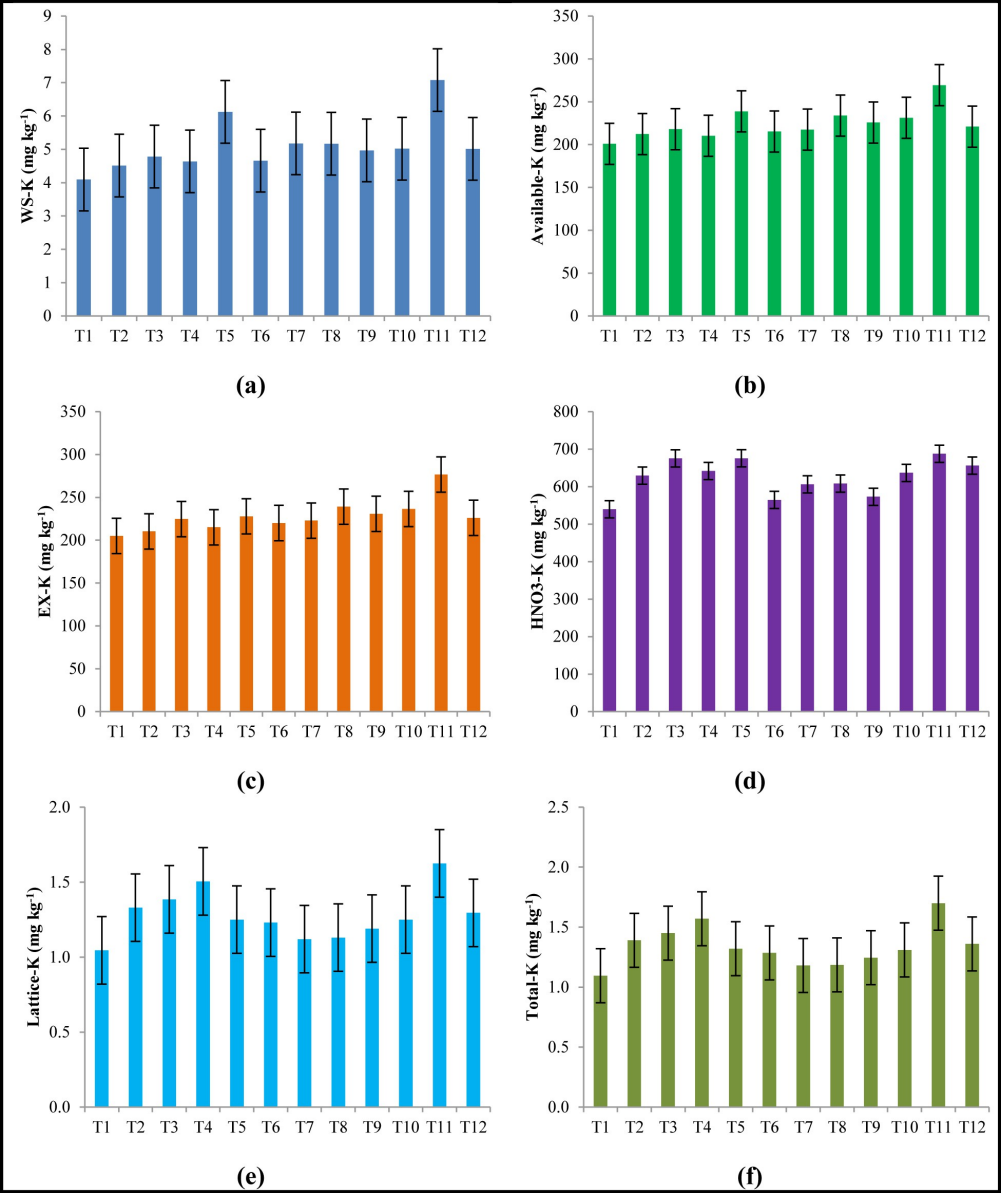
Effect of various INM modules on soil K fractions (a-Water soluble-K; b-Soil available-K; c-Exchangeable-K; d-HNO_3_-K; e-Lattice-K; f-Total-K; Error bars indicate critical difference at 5% level of significance; data is pooled of two years).

The data pertaining to the water soluble K, available K, exchangeable K, HNO_3_-K, lattice K and total K influenced by long term integrated nutrient management in 0–15 cm soil depth during 2012–13, 2013–14 and pooled of 2 years are presented in Tables 2 and 3 and Fig 1, respectively. In the year 2012–13 soil sample analysis value showed that INM module treatment 11 was obtained higher total K (1.142%), whereas 1.159% in the year 2013–14. This treatment having more amount of K fraction like water soluble, available K, exchangeable K, lattice K etc. Statistical analysis showed that most of the K fractions were significantly affected by INM module except lattice K and total K. The soil K fractions were observed in following order irrespective of treatments: total-K > Lattice-K > HNO_3_-K > Exchangeable-K > Available-K > Water soluble-K. In this experiment, different forms of K was affected by the application of INM modules under maize-chickpea cropping sequence. The water soluble K under various long- term INM modules ranged 3.71–8.08 mg kg^-1^ in 2012–13 and 4.48–6.08 mg kg^-1^ in the year 2013–14. The lowest concentration of water soluble K in present investigation might be attributed to the fact that the K in the soil solution is more easily utilized by the crop [17]. Sawarkar et al. [18] discussed the contributions of various potassium (K) fractions in soil, ranking them as follows: lattice-K > non-exchangeable-K > exchangeable-K > water-soluble-K. A similar trend in the abundance of K fractions in soils was also observed by Soremi et al. [19]. In a recent study, Jadhao et al. [20] reported the sequential dominance of K fractions in Vertisols of Akola as follows: lattice K > non-exchangeable K > exchangeable K > available K > water-soluble K. Earlier, Prakash and Singh [21] found the water-soluble K fraction to range from 8 to 50 mg kg^-1^ with a mean value of 18.25 for surface soil and 16.45 mg kg^-1^ for sub-surface soil.

**Table 2:** Effect of various INM treatments on soil K fractions in the year 2012–13.

Treatment	Water soluble-K	Available-K	Exchangeable-K	HNO_3_-K	Lattice -K	Total-K
	(mg kg^-1^)	(mg kg^-1^)	(mg kg^-1^)	(mg kg^-1^)	(%)	(%)
T_1_	3.71	207.5	203.8	513.7	1.039	1.090
T_2_	4.09	214.4	210.3	631.7	1.060	1.123
T_3_	4.66	228.3	223.7	682.7	1.072	1.141
T_4_	4.09	217.8	213.7	633.3	1.070	1.133
T_5_	6.25	246.1	239.8	635.3	1.056	1.120
T_6_	4.79	224.5	219.7	542.3	1.051	1.105
T_7_	4.98	225.7	220.7	551.0	1.050	1.105
T_8_	4.93	244.8	239.8	548.3	1.052	1.107
T_9_	4.97	241.5	236.5	532.3	1.054	1.107
T_10_	4.80	238.6	233.8	633.3	1.067	1.130
T_11_	8.08	281.3	273.2	623.0	1.079	1.142
T_12_	4.75	225.3	220.5	617.0	1.069	1.131
SEm ±	0.34	6.81	6.83	19.90	0.048	0.049
Lsd (p = 0.05)	1.00	19.99	20.03	58.36	NS	NS

**Table 3:** Effect of various INM treatments on soil K fractions in the year 2013–14.

Treatment	Water soluble-K	Available-K	Exchangeable-K	HNO_3_-K	Lattice -K	Total-K
	(mg kg^-1^)	(mg kg^-1^)	(mg kg^-1^)	(mg kg^-1^)	(%)	(%)
T_1_	4.48	202.5	198.0	565.0	1.044	1.100
T_2_	4.94	217.2	214.3	626.9	1.081	1.144
T_3_	4.91	219.5	212.3	667.5	1.083	1.150
T_4_	5.19	211.9	207.0	649.9	1.078	1.143
T_5_	6.00	243.8	237.8	715.8	1.078	1.150
T_6_	4.53	215.5	211.0	586.7	1.076	1.135
T_7_	5.38	219.9	214.5	661.1	1.071	1.137
T_8_	5.41	233.4	228.0	667.8	1.070	1.137
T_9_	4.97	220.0	215.0	613.4	1.071	1.132
T_10_	5.24	234.2	229.0	640.0	1.079	1.143
T_11_	6.08	271.8	265.7	751.9	1.084	1.159
T_12_	5.28	226.8	221.5	695.3	1.054	1.123
SEm ±	0.30	7.24	9.52	25.99	0.047	0.046
Lsd (p = 0.05)	0.88	21.24	27.91	76.24	NS	NS

### Water soluble K

The water soluble K under various long- term INM modules ranged 3.71 to 8.08 mg kg^-1^ in 2012–13 and 4.48 to 6.08 mg kg^-1^ in 2013–14. Water soluble K fraction was 3.71 in control plot and highest K level (8.08 mg kg^-1^) in treatment 2012–13 under treatment 11 of INM module (Table 3). Similarly, the average water soluble K across the treatments under study was found 5.10 mg kg^-1^. Similarly, Dhillon et al. [22] observed that the water-soluble K fraction constituted approximately 0.06% of the total K in some benchmark soil profiles of Punjab and Himachal Pradesh. In the surface soil (0–15 cm), the water-soluble K was found to be highest and statistically significant under the treatment receiving FYM at 20 t ha-1 for maize, followed by FYM at 5 t ha^-1^ (T11), in comparison to all the other treatments under study. Sharma and Verma [23] noted that the water-soluble K fraction increased by up to 45% when lantana was added as a source of K over time in acid Alfisols of Palampur. Pannu *et al*. [24] reported that incorporation of organic materials to the soil improved the water soluble K fraction over unamended control under long-term application of organic materials on different K fractions in an Alfisol under rice-wheat cropping system. Similarly, Majumdar *et al*. [25] also observed that water soluble-K increased significantly with increasing doses of K and addition of FYM. The results of water soluble K in surface soil are in conformity with the earlier findings of Sharma *et al*. [23], Mercykutty *et al*. [26], Sharma and Bhandari [27], Gurumurthy and Prakasha [28], Jatav and Dewangan [29] and Sharma and Paliyal [30].

### Available K

The soil available K under various long -term INM modules ranged 207.5–281.3 mg kg^-1^. Similarly, the average soil available K across the treatments under study was found 224.6 mg kg^-1^ (Fig 1). Although available K is represent only a small fraction of total K. Similarly, the treatment receiving FYM @ 20 t ha^-1^ to maize followed by FYM @ 5 t ha^-1^ (T_11_) showed highest soil available K and found statistically significant over rest all the treatments under study. The treatment with 100% STCR dose and/or with integration of organic manures showed significant with respect to the soil available K content. The balance application of K through the organic and inorganic sources of plant nutrients were contributing in most of the fractions during the study. Singh and Kuhad [31] was found as high as 0.61% of total K in different bio-climatic zones of Haryana. Prasad and Rajamannar [32] reported that K fertilizer application showed marked rise in available K in soils having slow and moderate K releasing capacity and these soils showed a decrease in available K with increasing crop maturity. Upadhyay and Bhandari [33] observed the available-K status (261.0 kg ha^-1^) of the soil increased with increase in fertilizer compared with control (177.4 kg ha^-1^) in an experiment in gravely loam Typic Hapludalf of Solan. Further, Arora and Chahal [34] also reported available K content varied from 41.5 to 314.0 mg kg^-1^ in the surface and 32.0 to 243.0 mg kg^-1^ in the subsurface soils with an average value of 76.0 mg kg^-1^. Similarly, Sharma and Bhandari [27] also reported available K content varied from 1.12 to 3.33 me 100 g^-1^ in the apple growing red soil. Majumdar *et al*. [25] reported increase in available K with increasing doses of K and FYM in a Hapludalf under rice -wheat cropping system. Similarly, Sawarkar *et al*. [18] also documented highest available-K under 100% NPK+FYM (295.2 kg ha^−1^), followed by 150% NPK (284.2 kg ha^−1^). The increase in available K upon the integrated application has already been reported by Kas *et al*. [35], Srinivasa Rao and Srinivas [36] and Meena *et al*. [37]. Incorporation of organic materials to the soil improved the available K fraction over unamended control and available K might be due to addition of K to available pool of the soil and mobilization of the native or non-exchangeable forms of K and charge the soil solution with K ions, resulting increase in the K availability in soil [24, 38].

### Exchangeable K

The exchangeable-K of a soil is difficult to define theoretically and to determine experimentally due to the absence of a sharp distinction between soluble and exchangeable fractions; and the existence of difficultly exchangeable K in some soils that is not immediately extracted by usual reagents. The exchangeable K concentration in soil was measured 203.8 mg kg^-1^ in the control plot and highest (273.2 mg kg^-1^) in the treatment 11 under INM module in the year 2012–13 (Table 2). Whereas, exchangeable K fraction was lower down in the year 2013–14 from 198.0 in control and 265.7 mg kg^-1^ treatment 11 of INM module, respectively. The exchangeable K under various long- term INM modules ranged 198.0–273.2 mg kg^-1^ in both the years. Similarly the average exchangeable K across the treatments under study was found 227.8 mg kg^-1^. Srivastava Rao *et al*. [39] reported 0.19 to 0.39 cmol kg^-1^ exchangeable K in the surface soil (0–15 cm) in vertisol with highest values was obtained in 100% NPK+FYM treatments. The treatment involving integrated nutrient management and/or recommended dose balanced fertilizers showed positive influence on exchangeable K and these treatments were found statistically significant over other treatments under study. Similarly, Jadhao *et al*. [20] also reported that the application of 100% NPK + FYM @ 5 t ha^-1^ significantly increased the exchangeable K 201 mg kg^-1^ in 0–15 cm depth soil. In practice, the determined exchangeable–K value is almost certain to be affected by one or more of these factors [40]. Exchangeable-K is the portion of the soil K that is electro- statically bound as an outer-sphere complex to the surfaces of clay minerals and humic substances. It is readily exchanged with other cations, also is readily available to soil, and plants [41]. The exchange adsorption of Ca and Mg relative to that of K and sodium is increased upon dilution of the system with water. Thus, neutral soil having soluble Ca and K, the dilution will result in an increase in exchangeable calcium and an equivalent decrease in exchangeable-K. Although there is no sharp distinction between exchangeable and water-soluble K for practical purposes the exchangeable-K is considered to be adsorbed on soil clay complexes and is replaced with neutral salt solutions like ammonium acetate in a relatively short time.

### HNO_3_-K, lattice K and total K

The soil HNO_3_-K under various long- term INM modules ranged 513.7 to 682.7 mg kg^-1^ in the year 2012–13; whereas 565–751.9 mg kg^-1^ in the year 2013–14. Similarly, the average soil HNO_3_-K across the treatments under study was found 624.4 mg kg^-1^. The treatment receiving FYM @ 20 t ha^-1^ to maize followed by FYM @ 5t ha^-1^ to chickpea (T_11_) showed highest soil HNO_3_-K and found statistically significant over rest all the treatments under study except in 2012–13 (Fig 1). The treatment receiving STCR dose (T_3_) found next superior treatment with respect to the soil HNO_3_-K. The value of lattice K under various long- term INM modules ranged 1.039 to 1.079% in the year 2012–13 and 1.044 to 1.084% in the year 2013–14; and the average lattice K across the treatments was found 1.28%. The lattice K found non significantly (p = 0.05) higher under the treatment receiving FYM @ 20 t ha^-1^ to maize followed by FYM @ 5 t ha^-1^ to chickpea (T11) over rest all the treatments under study (Fig 1). In this, experiment, total K content was non significantly affected by the different treatments. However, in both the years, total K was observed more under treatment T11 over rest of the treatments (Tables 2 & 3). Kori *et al*. [42] reported 1.334% as the average value of lattice-K in vertisol. Jadhao *et al*. [20] reported that the application of 100% NPK + FYM @ 5 t ha^-1^ significantly increased the lattice K (17078 mg kg^-1^) in 0–15 cm depth in Vertisol of central India. Ranganathan and Satyanarayana [43] reported that lattice K content followed a decreasing order in red, alluvium, laterite and black soils. The results of present investigation are in line with these findings. Total K refers to the sum of all the forms of K present in the soil. The total K content of the soil varied depending on the presence or absence of K bearing minerals and addition sources of K supply. The presence of minerals like feldspars (orthoclase and microcline), mica and illite enhances the total K in soil. Though the total K content provides an idea of the quantity of K present, it does not indicate anything about the quantity of K assessable for plant growth. In present investigation the total K under various long term INM modules ranged 1.10–1.70% and across the treatments it was found 1.34%. Dixit et al. [44] observed that this particular fraction varied from 185.48 to 2104.84 mg kg^-1^ in ten soil series in western Uttar Pradesh. In a study by Jadhao et al. [20], it was recorded that the application of 100% NPK + FYM at a rate of 5 tons per hectare significantly increased the non-exchangeable potassium content, reaching 945 mg kg-1 in the surface layer of Vertisols in Akola, Maharashtra. Gurumurthy and Prakasha [28] reported that the total potassium content in surface soils ranged from 0.77 to 1.59 percent. This form of potassium contributed the most to the total potassium content compared to other fractions, primarily because it includes potassium from potassium-bearing minerals. The treatment receiving FYM @ 20 t ha^-1^ to maize followed by FYM @ 5 t ha^-1^ to chickpea (T_11_) showed highest total K and found statistically significant over rest all the treatments under study. The treatment receiving STCR dose (T_3_) also found superior over other treatments (Tables 2 & 3). Dixit et al. [43] reported that the application of P and K decreased the total K content compared to the control group. This reduction was attributed to higher yields and increased nutrient uptake resulting from intensive cropping in a long-term fertilizer experiment conducted in Jabalpur, Madhya Pradesh. When a 100 percent optimal nitrogen dose was applied alone, it decreased the total K content even more than the control. This decrease was due to the development of residual acidity caused by the application of urea, which resulted in the release of non-exchangeable K into the soil solution. In contrast, Majumdar et al. [25] observed that the total K content remained almost unchanged despite the application of K fertilizer. It was also observed that addition of inorganic fertilizer and organic sources were minimized the K status in the Czech Republic soils [45].

## Conclusions

Potassium is an essential plant nutrient that plays a crucial role in determining crop yield and seed quality. Unfortunately, many farmers tend to overlook the importance of potassium application during crop production. It has been observed that most Indian soils have lower levels of potassium in the soil solution, which can limit crop yields. Integrated nutrient management (INM) offers a viable solution for maintaining soil fertility and enhancing crop yields. In this experiment, various INM modules were assessed to evaluate their impact on different potassium fractions in the soil. The results indicated that application of FYM at a rate of 20 tons per hectare per year in maize and 5 t ha^-1^ FYM (every years) in chickpea (T_11_) showed highest availability of K fractions in both the years. However, the treatment T_5_ (75% NPK of T3 + 5 t ha^-1^ FYM in maize and 100% P in chickpea) also significant improved the K fractions in soil. These approaches not only improved soil potassium fractions but also enhanced soil potassium availability. These findings are valuable for managing potassium in most soils, especially in maize-chickpea cropping sequences.
